# Dry immersion induced acute low back pain and its relationship with trunk myofascial viscoelastic changes

**DOI:** 10.3389/fphys.2022.1039924

**Published:** 2022-10-13

**Authors:** Anastasija Plehuna, David Andrew Green, Liubov E. Amirova, Elena S. Tomilovskaya, Ilya V. Rukavishnikov, Inessa B. Kozlovskaya

**Affiliations:** ^1^ King’s College London, Centre of Human & Applied Physiological Sciences, London, United Kingdom; ^2^ Laboratory of Gravitational Physiology of the Sensorimotor System, Institute of Biomedical Problems, Russian Academy of Sciences, Moscow, Russia; ^3^ Space Medicine Team, HRE-OM, European Astronaut Centre, European Space Agency, Cologne, Germany; ^4^ KBRwyle Laboratories GmbH, Cologne, Germany

**Keywords:** low back pain, muscle tone, myotonpro, dry immersion, space flight, myofascial tissue properties

## Abstract

Microgravity induces spinal elongation and Low Back Pain (LBP) but the pathophysiology is unknown. Changes in paraspinal muscle viscoelastic properties may play a role. Dry Immersion (DI) is a ground-based microgravity analogue that induces changes in m. *erector spinae* superficial myofascial tissue tone within 2 h. This study sought to determine whether bilateral m. *erector spinae* tone, creep, and stiffness persist beyond 2 h; and if such changes correlate with DI-induced spinal elongation and/or LBP.

Ten healthy males lay in the DI bath at the Institute of Biomedical Problems (Moscow, Russia) for 6 h. Bilateral lumbar (L1, L4) and thoracic (T11, T9) trunk myofascial tone, stiffness and creep (MyotonPRO), and subjective LBP (0-10 NRS) were recorded before DI, after 1h, 6 h of DI, and 30min post. The non-standing spinal length was evaluated on the bath lifting platform using a bespoke stadiometer before and following DI.

DI significantly modulated m. *erector spinae* viscoelastic properties at L4, L1, T11, and T9 with no effect of laterality. Bilateral tissue tone was significantly reduced after 1 and 6 h DI at L4, L1, T11, and T9 to a similar extent. Stiffness was also reduced by DI at 1 h but partially recovered at 6 h for L4, L1, and T11. Creep was increased by DI at 1 h, with partial recovery at 6 h, although only T11 was significant. All properties returned to baseline 30 min following DI. Significant spinal elongation (1.17 ± 0.20 cm) with mild (at 1 h) to moderate (at 6 h) LBP was induced, mainly in the upper lumbar and lower thoracic regions. Spinal length increases positively correlated (Rho = 0.847, *p* = 0.024) with middle thoracic (T9) tone reduction, but with no other stiffness or creep changes. Spinal length positively correlated (Rho = 0.557, *p* = 0.039) with Max LBP; LBP failed to correlate with any m. *erector spinae* measured parameters.

The DI-induced bilateral m. *erector spinae* tone, creep, and stiffness changes persist beyond 2 h. Evidence of spinal elongation and LBP allows suggesting that the trunk myofascial tissue changes could play a role in LBP pathogenesis observed in real and simulated microgravity. Further study is warranted with longer duration DI, assessment of IVD geometry, and vertebral column stability.

## 1 Introduction

Low back pain (LBP) is the most common musculoskeletal condition affecting adults worldwide (Balagué et al., 2012). Typically, additional loading exaggerates LBP, although in the majority of individuals the specific aetiology of LBP cannot be identified (Hartvigsen et al., 2018). Acute exposure to microgravity is also associated with LBP (Kertsman et al., 2012), and whilst its aetiology is also unknown, the absence of diurnal gravitational loading on the spine presumably plays a key role (Sayson et al., 2013). In addition to LBP, microgravity induces stature increments of up to 6 cm evident in the first few days of flight (Brown, 1977). Such increments can cause operational issues including difficulties donning an Extra-Vehicular Activity (EVA) suit or fitting into the pre-moulded “Kazbek” seat pan of the Russian Soyuz capsule (Rajulu and Benson, 2009).

Microgravity-induced changes to the spinal column may also increase the risk of spinal injury due to the loading generated during in-orbit exercise on the International Space Station (ISS), descent, and landing, in addition to activity in the post-flight rehabilitation phase (Green and Scott, 2018). Such risks pose a significant health concern for space exploration missions and in particular those to the Lunar or Martian surface (Pool-Goudzwaard et al., 2015).

Astronauts frequently report LBP in the hours following insertion into microgravity that can persist for up to 4 weeks (Wing et al., 1991; Kertsman et al., 2012). The majority of astronauts (86%) report lumbar pain, although thoracic (12%) and cervical pain (2%) have also been noted (Kertsman et al., 2012). Whilst the pathophysiology of microgravity-induced LBP is unknown, supra-physiological swelling of intervertebral discs (IVD) has been suggested (Sayson et al., 2013; Belavý et al., 2016; Robin, et al., 2022). Indeed, a recent in-flight ultrasound evaluation of IVDs revealed disk desiccation, osteophytes, and qualitative changes in angle and height (Garcia et al., 2018). A further on-ground experiment has revealed an increase in IVD and elevated apparent diffusion coefficient (Treffel et al., 2020). However, magnetic resonance imaging identified no changes in IVD geometry in six NASA astronauts from pre-flight, 1–2 days, and again 2 months post-landing (Chang et al., 2016) although changes in IVD endplates and impaired spinal segment kinematics are potentially related to trunk muscle atrophy may contribute to post-flight IVD herniation risk (Bailey et al., 2018; Bailey et al., 2022).

Whilst microgravity-induced declines in muscle power, strength, and tone secondary to muscle atrophy are observed in the lower limbs (Kozlovskaya, 2007) and in paraspinal (anti-gravity) muscles (Schneider et al., 2015; Rukavishnikov et al., 2017; Amirova et al., 2021) such changes cannot underlie the rapid onset of LBP (Green and Scott, 2018). However, acute changes in paraspinal muscle and superficial myofascial tissue biomechanical and viscoelastic properties may potentially precipitate microgravity-induced LBP.

For instance, on Earth chronic LBP sufferers show a significant reduction in lower back muscle and myofascial tone (Nair et al., 2016). In fact, changes in lumbar myofascial tissue tone correlate with chronic LBP (Haladaj and Topol, 2016). Interestingly, research performed by teams from the Institute of Biomedical Problems (IBMP) in Moscow (Russia) provided evidence of rapid muscle tone change termed hypogravitational muscle syndrome following gravitational unloading (Grigor’ev et al., 2004; Rukavishnikov et al., 2017). Reductions in tone and stiffness—have also been reported in several lower limb muscles e.g., m. *triceps surae*: m. *gastrocnemius lateralis* and *medialis*, and m. *soleus* (Nemirovskaya and Shenkman, 2002; Kozlovskaya, 2007; Miller et al., 2012). Such changes may be triggered by the absence of stimuli that in gravity activate postural muscle activity (Kozlovskaya, 2007; Shenkman et al., 2021).

Indeed, a recent parabolic flight revealed rapid declines in the transverse tone of m. *erector spinae* (Schneider et al., 2015). Similar declines have been suggested to play a role in the development of LBP on Earth (Sung et al., 2009). Conceivably, acute reductions in paraspinal tone may contribute to IVD swelling (Chang et al., 2016) and/or spinal curvature flattening (Lee et al., 2003) and thereby spinal elongation (Rukavishnikov et al., 2017), vertebral column instability (Belavý et al., 2016) and LBP (Amirova et al., 2021). Furthermore, creep, a viscoelastic property that describes progressive elongation of tissue under tensile stress (Schneider et al., 2015) may also provide insight into changes that contribute to microgravity-induced spinal column elongation and LBP (Abboud et al., 2016).

Unfortunately, spinal assessment on the ISS has largely been restricted to pre- and post-flight measures (e.g., Chang et al., 2016; Bailey et al., 2022). Furthermore, whilst head-down tilt bed rest (HDTBR) is the most commonly employed ground-based microgravity analogue (Hargens and Vico, 2016; Vico and Hargens, 2018), its ability to induce back pain (Hutchinson et al., 1995), and other spinal column changes associated with spaceflight appears limited (Green and Scott, 2018). For instance, increments in spinal length after 3 days of HDTBR (Belavý et al., 2011) did not differ from 8 h of regular sleep on Earth (Styf et al., 1997).

Dry Immersion (DI) is an alternative ground-based microgravity analogue that involves the removal of mechanical support (supportlessness) that was developed by IBMP in the 1960s (Shulzhenko and Vil-Vilyams, 1975). DI involves the subject being “immersed” in a water-filled bath whilst laying on a waterproof flexible material allowing them to remain dry while being freely suspended (Tomilovskaya et al., 2019). DI has been reported to rapidly induce LBP of similar nature and intensity to that induced in microgravity (Rukavishnikov et al., 2017; Tomilovskaya et al., 2019). As a result, DI has been proposed as a potentially more valid analogue of microgravity-induced LBP (Kozlovskaya, 2008; Navasiolava et al., 2011; Tomilovskaya et al., 2019).

DI via resonance vibrography has been demonstrated to induce a rapid change in myofascial tissue properties, including a bilateral decrease in back extensor tone (m. *longissimus dorsi* in the projection of L2–L3) (Rukavishnikov et al., 2017). Furthermore, a rapid bilateral drop in m. *erector spinae* (in T9-T8, T12-L1, and L3-L4 cord projection) and trapezius muscle tone were recently observed during up to 2 h of DI (Amirova et al., 2021). However, whether such changes persist, and are correlated with LBP is unclear. In addition, asymmetry of paraspinal (Danneels et al., 2000; Hides et al., 2008) and pelvic (Levangie, 1999) muscle biomechanical and viscoelastic properties have been suggested to be a potential factor underlying LBP aetiology and prognosis on Earth (Hodges et al., 2006).

Amirova and co-workers (2021) employed handheld myotonometry (MyotonPRO) which has been used in parabolic flight (Schneider et al., 2015), HDTBR (Schoenrock et al., 2018), and DI (Treffel et al., 2016; de Abreu et al., 2017; Rukavishnikov et al., 2017). The MyotonPRO is reported to provide reliable paraspinal (Hu et al., 2018) resting myofascial tissue tone assessment (Kelly et al., 2018; Muckelt et al., 2022) via analysis of induced tissue oscillations (Lo et al., 2017). The MyotonPRO also provides indices of dynamic stiffness and creep (Simons and Mense, 1998). However, these measures were not reported by Amirova and co-workers (2021), although increased muscle stiffness was observed in m. *erector spinae* and m. *lumbar multifidus* following 60-days HDTBR (Schoenrock et al., 2018). Thus, this study aimed to determine whether bilateral m. *erector spinae* tone, in addition to creep and stiffness, persist beyond 2 h, and if such changes are associated with DI-induced spinal elongation and/or LBP.

## 2 Methods

Ten healthy males (Table 1) provided written informed consent to participate in the study that received ethical approval from the Physiological Section of the Biomedicine Ethics Committee at the Institute of Biomedical Problems, a part of the Russian Academy of Science (IRB protocol #401, 15.07.2015). All subjects reported no musculoskeletal (including spinal) disorder prior to inclusion in the study.

**TABLE 1:** Subjects’ body composition data.

Subject	Age	Weight (kg)	Height (cm)	BMI
1	26	65.3	178	20.60
2	25	70.3	175	23.00
3	20	78.5	177	25.00
4	20	73.5	179	22.90
5	23	62.4	176	20.10
6	27	57.8	172	19.50
7	30	56.4	174	18.60
8	26	74.8	173	24.90
9	27	60.5	175	19.80
10	20	71.2	177	22.70
Mean	24.40	67.07	175.60	21.71
SEM	0.92	2.08	0.57	0.63

Each participant attended the laboratory at the Institute of Biomedical Problems (Moscow, Russia) on a single occasion, where they lay for 6 h in the DI bath. Lumbar and thoracic trunk myofascial tone, stiffness, and creep, as well as subjective LBP, were recorded 2 h before (PRE), after 1 (1 h DI) and 6 h (6 h DI) of DI, and 30 min after (RECOVERY) being raised out of DI. The non-standing spinal length was evaluated before (PRE) and following (POST) DI.

**FIGURE 1:**
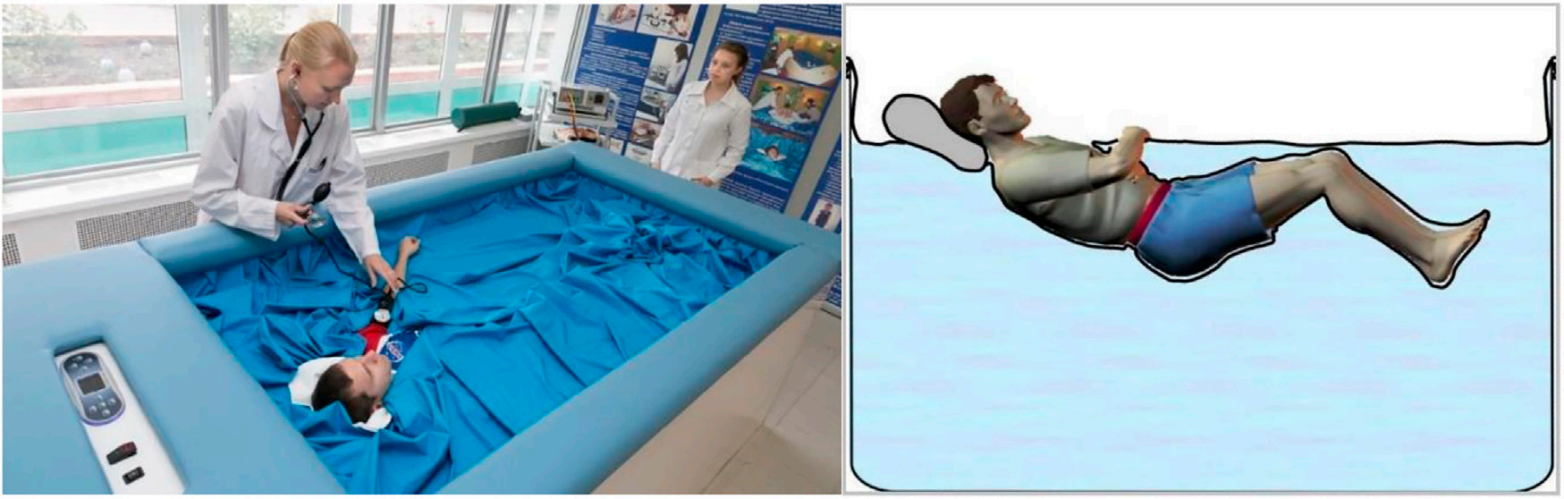
Left: Subject immersed in the Dry Immersion (DI) bath being separated from the water by non-elastic waterproof fabric (Image credit: IBMP press secretary Oleg Voloshin). Right: Schematic representation of the body posture in DI (Shulzhenko and Vil-Villiams, 1975).

Subjects were passively lowered into and raised out of the DI water-filled bath (210 × 90 × 110 cm) covered with a non-elastic waterproof fabric on an in-built lifting platform (Figure 1). The water was maintained at a comfortable thermoneutral temperature of 32.5°C ± 2°C, with an ambient temperature of 24°C–26°C. Subjects were requested to remain supine, relaxed, and as still as possible throughout testing.

**FIGURE 2:**
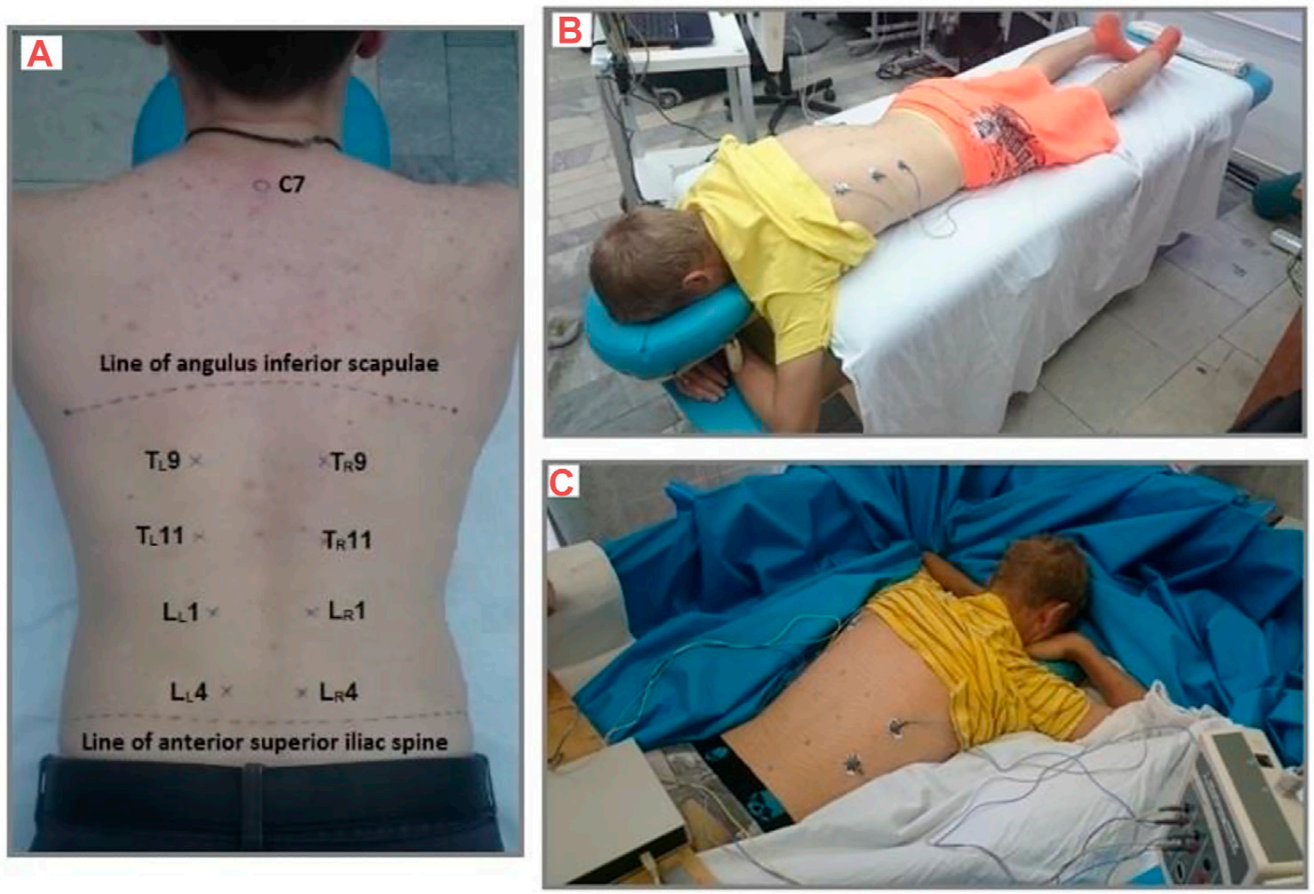
Measurement of Lower Lumbar (LL4, LR4), Upper Lumbar (LL1, LR1), Lower Thoracic (TL11, TR11), and Middle Thoracic (TL9, TR9) locations. **(A)** Subject remained prone for lumbar and thoracic trunk myofascial tissue assessment **(B)** on a couch, and **(C)** in the DI bath.

Bilateral viscoelastic properties of lumbar and thoracic trunk myofascial tissues (Lower Lumbar (LL4, LR4), Upper Lumbar (L_L_1, L_R_1), Lower Thoracic (T_L_11, T_R_11), and Middle Thoracic (T_L_9, T_R_9) regions (Figure 2A) were assessed using a small handheld MyotonPRO (Myoton AS, Estonia) with subjects in the prone position with arms under the forehead, on a couch for PRE and POST DI recordings (Figure 2B). Assessment during DI required temporary lifting of the subjects with the in-built platform to allow passive turning to prone position (Figure 2C) by a team of investigators before immediately being turned back and returned to the supine DI position. Measurement points were marked on the skin above the muscle belly to ensure recording repeatability.

The MyotonPRO applies a mechanical impulse to the tissue at the application point with the probe placed perpendicularly to the skin. Consistent generation of approx. 0.18 N pre-load—to ensure subcutaneous tissue pre-compression—activates a series of five short (15 ms) 0.4 N impulses of the instrumented actuator. The following tissue properties were calculated: tone (natural oscillation frequency, Hz), biomechanical properties—dynamic stiffness (N/m), and viscoelastic properties—creep (ratio of deformation with relaxation time: Deborah Number) (Schneider et al., 2015).

The recordings were obtained at rest (confirmed by real-time EMG recording <0.1 mV (Neuro-MEP-4 device, Russia) of the measurement site following subjects being instructed to exhale normally and then hold their breath around their Functional Residual Capacity ***.***

**FIGURE 3:**
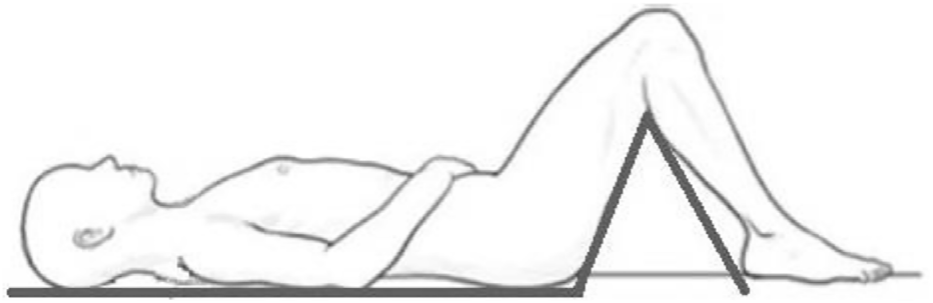
Body position on a bespoke stadiometer device.

Spinal length (distance from cranium vertex to the point where m. gluteus maximus abuts to the angled hip support) was measured when on the DI bath lifting platform using a bespoke stadiometer (specifically designed and made for the current experiment by Dr. I. Rukavishnikov, Figure 3). The stadiometer is designed to replicate an astronaut’s body position in a Soyuz “Kazbek” seat pan whilst also eliminating axial gravitational loading.

DI-induced subjective back pain was assessed via a body map for pain localization with a numeric Rating Scale (NRS: 0–10 severe pain) employed to evaluate pain intensity (Hawker et al., 2011).

All physiological data were normally distributed (Kolmogorov—Smirnov test). The effect of laterality (Left vs. Right side) on DI-induced trunk myofascial viscoelastic properties was assessed with a two-way ANOVA during DI (PRE, 1 h DI, 6 h DI, RECOVERY). As no effect of laterality was observed, data from both sides were pooled for each location and the effect of time (DI exposure) was evaluated by a one-way ANOVA, with post-hoc Bonferroni corrected t-tests.

The effect of 6 h DI on spinal length was evaluated using paired t-tests (PRE vs. RECOVERY). DI effects on maximum pain at 1 h DI, 6 h DI, and RECOVERY were evaluated using Wilcoxon signed-rank testing.

The relationship between changes in trunk myofascial tissue tone, stiffness, creep, spinal length, and maximum-induced pain intensity was assessed via non-parametric Spearman’s correlation (Rho).

All data is represented as Mean ± Standard Error Mean (SEM). All statistical analysis was performed using the Prism8 software (GraphPad Software, Inc. United States) with statistical significance defined as *p* < 0.05.

## 3 Results

As trunk myofascial tissue tone, stiffness and creep did not significantly differ between the left and right recordings they were averaged at all levels.

**FIGURE 4:**
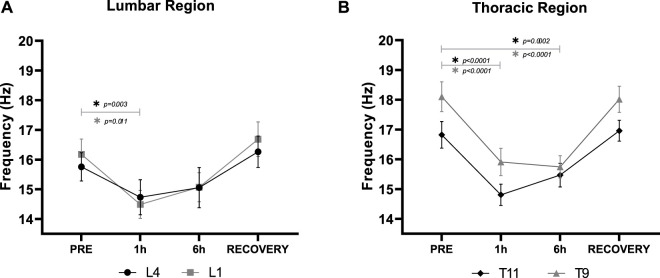
Mean (±SEM) bilateral m. *erector spinae* myofascial tone (Hz) in the **(A)**: Lower Lumbar (L4) and Upper Lumbar (L1), and **(B)**: Lower Thoracic (T11), and Middle Thoracic (T9) regions before (PRE), during Dry Immersion (DI) after 1 (1 h) and 6 (6 h) hours, and 30 min after being raised out of DI (RECOVERY). *Statistically significant difference (*p* < 0.05) between time points (post-hoc Bonferroni corrected *t*-tests).

Mean bilateral m. *erector spinae* myofascial tissue tone demonstrated a similar trend across all locations (Figure 4). Mean Lower Lumbar (L4) myofascial tone was significantly (F_3,27_ = 14.12, *p* < 0.001) reduced by DI (Figure 4A). Myofascial tone (*p* = 0.003) declined by 6.5% ± 0.5% after 1 h DI. However, Lower Lumbar myofascial tone was not significantly (*p* = 0.070) lower than PRE at 6 h DI with no difference between 1 and 6 h DI (*p* > 0.999). RECOVERY Lower Lumbar myofascial tone did not differ from PRE (*p* = 0.360).

Similarly, Upper Lumbar (L1) myofascial tone was significantly (F_3,27_ = 8.48, *p* < 0.001) reduced by DI (Figure 4A). Myofascial tone significantly (*p* = 0.010) declined by 10.4 ± 0.1% after 1 h DI. However, Upper Lumbar myofascial tone was not significantly (*p* = 0.194) lower than PRE at 6 h, nor between 1 and 6 h DI (*p* > 0.999). RECOVERY Upper Lumbar myofascial tone did not differ from PRE (*p* > 0.999).

Lower Thoracic (T11) myofascial tone was significantly (F_3,27_ = 30.08, *p* < 0.001) reduced by DI (Figure 4B). Myofascial tone significantly (*p* < 0.001) declined by 12.0% ± 0.7% after 1 h DI and by 8.0% ± 0.01% after 6 h (*p* < 0.001). No difference between 1 and 6 h DI (*p* > 0.999) was observed. RECOVERY Lower Thoracic myofascial tone did not differ from PRE (*p* > 0.99).

Middle Thoracic Region (T9) myofascial tone was significantly (F_3,27_ = 68.07, *p* < 0.001) reduced by DI (Figure 4B). Myofascial tone significantly (*p* < 0.001) declined by 12.1% ± 0.5% after 1 h, and by 13.0% ± 0.2% (*p* < 0.001) after 6 h of DI. No difference between 1 and 6 h DI (*p* > 0.999) was observed. RECOVERY Middle Thoracic myofascial tone did not differ from PRE (*p* > 0.999).

**FIGURE 5:**
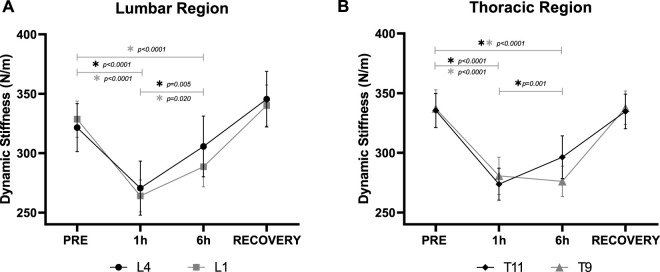
Mean (±SEM) bilateral m. *erector spinae* myofascial dynamic stiffness (N/m) in the **(A)**: Lower Lumbar (L4) and Upper Lumbar (L1), and **(B)**: Lower Thoracic (T11), and Middle Thoracic (T9) regions before (PRE), during Dry Immersion (DI) after 1 (1 h) and 6 (6 h) hours, and 30 min after being raised out of DI (RECOVERY). *Statistically significant difference (*p* < 0.05) between time points (post-hoc Bonferroni corrected *t*-tests).

Mean bilateral m. *erector spinae* myofascial tissue stiffness demonstrated somewhat similar trends across all locations. Lower Lumbar (L4) myofascial stiffness was significantly (F_3,27_ = 14.2, *p* < 0.001) reduced by DI (Figure 5A). Myofascial stiffness significantly (*p* < 0.001) declined by 16.6% ± 2.8% after 1 h DI. However, there was a partial recovery at 6 h DI which was significantly (*p* = 0.005) higher than 1 h DI, and not significantly differ from PRE (*p* = 0.600). RECOVERY Lower Lumbar myofascial stiffness did not differ from PRE (*p* = 0.100).

Upper Lumbar (L1) myofascial stiffness was significantly (F_3,27_ = 43.53, *p* < 0.001) reduced by DI (Figure 5A). Myofascial stiffness significantly (*p* < 0.001) declined by 19.6% ± 2.0% after 1 h, and by 12.3% ± 2.8% (*p* < 0.001) after 6 h DI, although the latter was significantly (*p* = 0.020) weaker. RECOVERY Upper Lumbar myofascial stiffness did not differ from PRE (*p* = 0.850).

Lower Thoracic (T11) myofascial stiffness was also significantly (F_3_,_27_ = 68.52, *p* < 0.001) reduced by DI (Figure 5B). Myofascial stiffness significantly (*p* < 0.001) declined by 18.5% ± 1.3% after 1 h, and by 12.2 ± 2.0% (*p* < 0.001) after 6 h DI, although the latter was significantly (*p* = 0.001) weaker. RECOVERY Lower Thoracic myofascial stiffness did not differ from PRE (*p* > 0.999).

Middle Thoracic (T9) myofascial stiffness was significantly (F_3,27_ = 75.36, *p* < 0.001) reduced by DI (Figure 5B). Myofascial stiffness significantly (*p* < 0.001) declined by 16.9% ± 1.5% after 1 h (*p* < 0.001) and 18.0% ± 1.3% (*p* < 0.001) after 6 h DI. No difference between 1 and 6 h DI (*p* > 0.999) Middle Thoracic stiffness was observed, and RECOVERY did not differ from PRE (*p* > 0.999).

**FIGURE 6:**
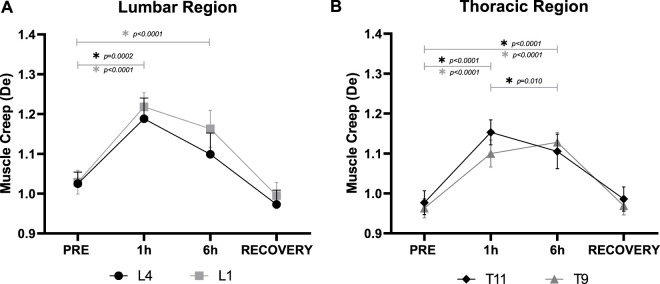
Mean (±SEM) bilateral m. *erector spinae* myofascial creep (De) in the **(A)**: Lower Lumbar (L4) and Upper Lumbar (L1), and **(B)**: Lower Thoracic (T11), and Middle Thoracic (T9) regions before (PRE), during Dry Immersion (DI) after 1 (1 h) and 6 (6 h) hours, and 30 min after being raised out of DI (RECOVERY). *Statistically significant difference (*p* < 0.05) between time points (post-hoc Bonferroni corrected *t*-tests).

Mean bilateral m. *erector spinae* myofascial creep demonstrated a similar trend across all locations. Lower Lumbar (L4) myofascial tissue creep was significantly (F_3,27_ = 16.87, *p* < 0.001) increased by DI (Figure 6A). Creep significantly (*p* < 0.001) increased by 15.8% ± 3.3% after 1 h DI which was weaker at 6 h and thus not significantly different from PRE (*p* = 0.17) whilst tending to be lower than 1 h DI (*p* = 0.060). RECOVERY Lower Lumbar creep did not differ from PRE (*p* = 0.710).

Upper Lumber (L1) myofascial creep was significantly (F_3,27_ = 37.47, *p* < 0.001) increased by DI (Figure 6A). Myofascial creep significantly (*p* < 0.001) increased by 18.7% ± 2.8% after 1 h, that was 13.0% ± 3.0% (*p* < 0.001) after 6 h DI although they did not differ between 1 and 6 h (*p* = 0.200). RECOVERY Upper Lumber myofascial creep did not differ from PRE (*p* > 0.999).

Lower Thoracic (T11) myofascial creep was also significantly (F_3,27_ = 75.28, *p* < 0.001) increased by DI (Figure 6B). Myofascial creep significantly (*p* < 0.001) increased by 18.1% ± 1.2% after 1 h, that was 12.8% ± 1.7% (*p* < 0.001) after 6 h DI leading to a significant (*p* = 0.010) difference between 1 and 6 h DI. RECOVERY Lower Thoracic creep did not differ from PRE (*p* > 0.999).

Middle Thoracic (T9) creep was also significantly (F_3,27_ = 61.02, *p* < 0.001) increased by DI (Figure 6B). Creep significantly (*p* < 0.001) increased by 14.1% ± 1.7% after 1 h, and 17.4% ± 1.5% (*p* < 0.001) after 6 h DI although no difference (*p* = 0.500) between 1 and 6 h DI. RECOVERY Middle Thoracic creep did not differ from PRE (*p* > 0.999).

All participants exhibited spinal elongation resulting in a significant (*p* < 0.001) mean spinal length increase after 6 h DI (POST-PRE) (Table 2).

**TABLE 2:** Individual and mean (±SEM) (Δ) change in spinal length Post-Pre 6 h Dry Immersion (DI). * Indicates significant paired *t*-test (*p* < 0.001).

Subject number	Max. change (Δ) in spinal length (cm)	Percentage difference (%)
1	0.2	0.1
2	0.5	0.3
3	1.5	0.8
4	1.0	0.6
5	1.0	0.6
6	1.5	0.9
7	0.4	0.2
8	1.5	0.9
9	2.4	1.4
10	1.7	1.0
Mean	1.2*	**0.7**
SEM	**0.2**	**0.1**

Bold values are representing mean ±SEM change in spinal length (cm) and mean ±SEM percentage difference (%).

**FIGURE 7:**
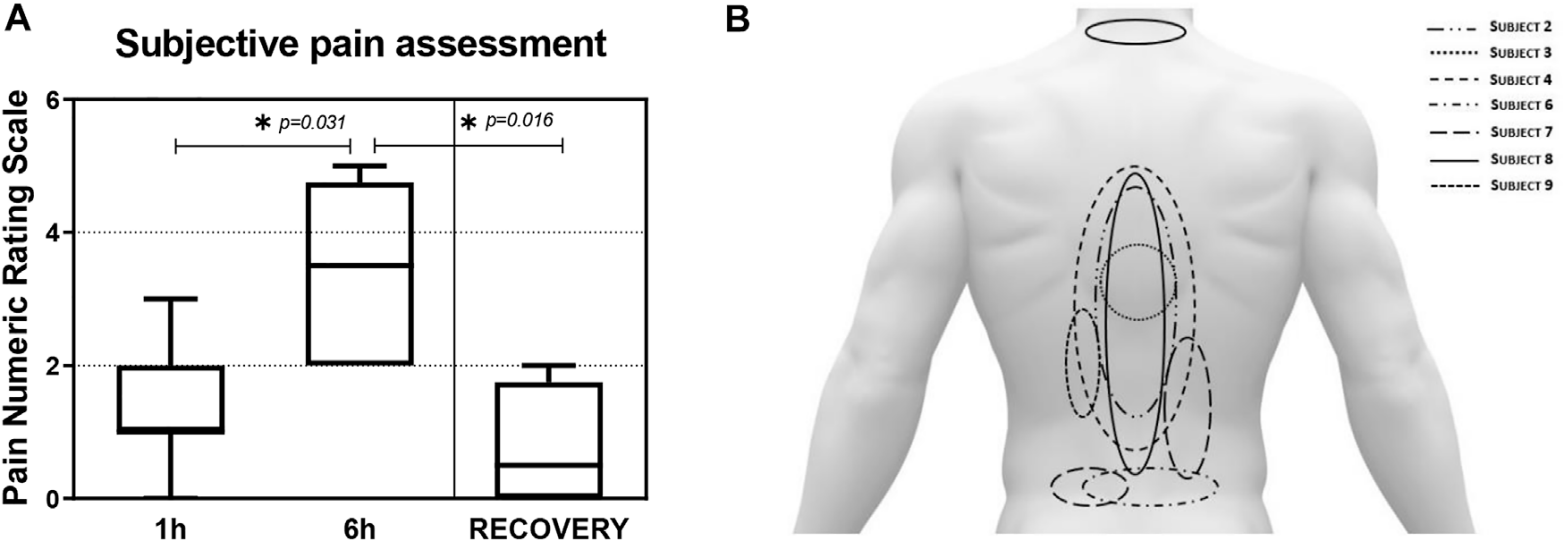
**(A)**: Mean (±SEM) back pain intensity (Numeric Rating Scale: 0–10: Max) scores after 1 (1 h), 6 h (6 h) Dry Immersion (DI), and 30 min after DI (RECOVERY). *Statistically significant (*p* < 0.05) Wilcoxon signed-rank test. **(B)**: Subjective back pain locations as drawn by the seven (out of ten) subjects that reported back pain after 6 h DI.

No subject reported back pain prior to DI (PRE). Seven out of ten subjects reported back pain (1.4 ± 0.9) after 1 h DI that was significantly (*p* = 0.031) higher at 6 h (3.4 ± 1.3) vs. 1 h DI (Figure 7A). The highest pain rating was 5 (equating to ‘moderate pain’ on the 0-10 NRS) reported by two subjects. Back pain was significantly (*p* = 0.016) ameliorated 30 min after DI (RECOVERY) compared to 6 h DI.

The seven subjects who reported back pain stated that it radiated on both sides of the spine (Figure 7B). Most subjects reported pain in the Upper Lumbar and Lower Thoracic regions, with only one subject reporting pain in the Cervical region. No radiating of pain to the gluteal region or lower limbs was reported.

**FIGURE 8:**
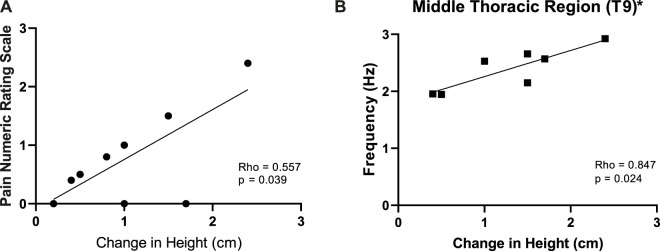
Relationship between the changes in spinal height (Post-Pre) (cm) vs. back pain **(A)** and changes in Middle Thoracic (T9) m. *erector spinae* tone (Hz) **(B)** induced by 6 h Dry Immersion (DI). * Statistically significant Spearman’s correlation (Rho).

Spinal elongation positively correlated with Maximal LBP (Figure 8A). Spinal elongation and Maximal LBP failed to correlate with any changes in m. *erector spinae* viscoelastic properties except for Middle Thoracic region. Spinal length increases induced by 6 h DI positively correlated (Rho = 0.847; *p* = 0.024) with Middle Thoracic (T9) myofascial tone reduction (Figure 8B).

## 4 Discussion

The main findings of the study were that DI significantly modulated m. *erector spinae* myofascial tissue biomechanical and viscoelastic properties at L4, L1, T11, and T9 with no effect on laterality. The bilateral myofascial tone was reduced after 1 and 6 h DI to a similar extent. Stiffness was also reduced by DI at 1 h but partially recovered at 6 h for L4, L1, and T11. Creep was increased by DI at 1 h, which also tended to partially recover at 6 h, although only T11 was significant. All measured parameters returned to baseline levels following DI. Significant (albeit mild) spinal elongation was induced by 6 h DI in all subjects, with the majority reporting LBP, mainly in the upper lumbar and lower thoracic regions that were mild at 1 h but increased to moderate after 6 h DI. Spinal length increases correlated with middle thoracic (T9) myofascial tone reduction, but with no other viscoelastic changes. Spinal length positively correlated with max LBP, but LBP failed to correlate with any m. *erector spinae* measured parameters.

### 4.1 Changes in m. *erector spinae* myofascial tissue properties

No effect of laterality (i.e., asymmetry) was observed on m. *erector spinae* viscoelastic properties recorded via myotonometry with the MyotonPRO. This contrasts with the asymmetry reported in paraspinal (Danneels et al., 2000; Hides et al., 2008) and pelvic (Levangie, 1999) muscle viscoelastic properties proposed to contribute to LBP on Earth (Hodges et al., 2006). However, as the DI exposure was only 6 h and only 10 subjects were tested further study is warranted.

DI significantly reduced bilateral m. *erector spinae* tone at 1 and 6 h to a similar extent—consistent with rapid declines in the transverse tone of m. *erector spinae* induced by acute (<22 s) unloading (Schneider et al., 2015). The study also extends findings of rapid bilateral reductions in m. *erector spinae* at the T9-T8, T12-L1, and L3-L4 cord projections and trapezius myofascial tone during up to 2 h of DI (Amirova et al., 2021).

Bilateral m. *erector spinae* stiffness was also reduced by DI at 1 h, but that partially recovered at 6 h for L4, L1, and T11. Thus, the present study suggests that short exposure to DI can trigger an acute reduction in myofascial tissue tone and stiffness, indicating possible changes in motor neuron activation patterns, and suppression of tonic motor neuron activities (Kozlovskaya and Kirenskaya, 1987; Kozlovskaya, 2002; Kozlovskaya et al., 2006; Shenkman et al., 2007), sensory and motor ataxia (Tomilovskaya et al., 2014; Shigueva et al., 2015), and significant hypersensitivity of the proprioceptive pathways (Grigor’ev et al., 2004).

Reductions in m. *erector spinae* stiffness contrast with increases observed in m. *erector spinae* and m. *lumbar multifidus* following 60-days HDTBR (Schoenrock et al., 2018). However, HDTBR is not associated with LBP (see Green and Scott, 2018). Thus, the DI-induced changes in myofascial tone may relate to the withdrawal of support (Kozlovskaya et al., 2007) and may be associated with LBP development. However, LBP was increased at 6 vs. 1 h, in contrast to attenuation of stiffness reductions at L4, L1, and T11. Yet, it may be that differential changes in stiffness around the vertebral column contribute to the development of LBP (Sung et al., 2009).

According to Grigor’ev and co-workers (Grigor’ev et al., 2004), DI promotes partial ‘deafferentation’ of the nervous system due to the withdrawal of support stimuli, accompanied by reduced muscle proprioceptive input. An association between muscle tone changes and support-related afferentation has been observed in the lower limbs (Kozlovskaya et al., 1984; Kozlovskaya, 2002) and our data suggests similar effects may be manifest in trunk musculature.

Myofascial creep was increased by DI at 1 h, that also tended to partially recover at 6 h, although only T11 was significant. To the authors’ knowledge, this study is the first to examine the association of LBP induced by support unloading with myofascial creep. The increase in viscoelastic tissue creep could be partially explained by the opposition of gravitational force withdrawal (Guo, 2002; Solomonow et al., 2003) and the diminishing protection from spine instability (Solomonow, 2012; Treffel et al., 2017).

DI induces a “flexed” spinal curvature similar to that observed in microgravity (NASA–STD-3000, 2014). As the pelvis is relatively heavy, it tends to sink during DI; the thorax, in contrast, tends to rise due to the buoyancy of the chest cavity and the fact that the head and neck are elevated on a pillow. Despite this, the withdrawal of gravitational loading reduces axial loads on the torso, and may significantly modulate lumbar spine stabilization by eliciting viscoelastic changes in paraspinal muscles (LeBlanc et al., 1994). Furthermore, an increase in myofascial creep could be associated with activation of the trunk muscles in response to viscoelastic changes, which could modulate dynamic intervertebral disc strain (Hayward and Dolan, 2018) that can precipitate LBP development. However, further study is required to investigate this hypothesis.

Interestingly, 30 min after DI all measured parameters recovered to PRE levels—confirming that whilst DI induces viscoelastic and biomechanical changes, they are rapidly reversible. Such findings support the proposition that post-flight testing should be rapid—to avoid failure to observe significant spinal geometric changes (Chang et al., 2016) despite small increases in IVD height observed in flight (Garcia et al., 2018), due to recompression (Green and Scott, 2018).

It is important to acknowledge that MyotonPRO is a highly sensitive device whose measurement accuracy may be affected by the differences in subjects’ positions. The current study has tried to maintain a similar position of the subjects through all measurement stages whenever possible, although the positions of their hand on the couch and in the bath differed slightly due to bath design.

### 4.2 Spinal elongation

Whilst significant albeit mild (1.2 ± 0.2 cm) spinal elongation was induced by 6 h DI. However, the largest increase in a single participant was 2.4 cm. Such increments, whilst substantial are lower than some spinal length changes reported inflight (i.e., 1.5–6.1 cm; Brown, 1977; Kimura et al., 2001; Kertsman et al., 2012; Young and Rajulu, 2020). Whilst the underlying mechanisms are unknown spinal elongation may be associated with a reduction of lumbar and thoracic curvature, and/or an increase in vertebral disc height (Sayson & Hargens, 2008; Sayson et al., 2013; Belavy et al., 2016). Declines in postural myofascial tissue tone and stiffness may potentially promote increases in IVD height. Whether this is in a case in DI warrants further study.

### 4.3 Low back pain

6 h of DI induced LBP in 7 out of 10 participants, mainly in the upper lumbar and lower thoracic regions that was mild at 1 h but increased to moderate at 6 h. The LBP was described as dull and deep, which are prominent features of pain originating from lower lying tissues (Mannion et al., 2007) not assessed in this study. During DI, visceral mass is drawn towards the diaphragm muscle, which may explain the prevalence of pain in the lumbar region and visceral subcostal pain. Moreover, the flexed lower limb position in DI can lead to retroversion of the pelvis, which could facilitate the decrease in lumbar curvature (Treffel et al., 2016).

Data obtained from previous DI experiments (3–21 days) demonstrated that back pain sensations - described as blunt aching, mainly in the upper lumbar spine region (Rukavishnikov et al., 2017; Tomilovskaya et al., 2020) were observed during the first 36 h in DI (Rukavishnikov et al., 2017; Treffel et al., 2017)—again broadly consistent with that reported by some crew (Pool-Goudzwaard et al., 2015) during early spaceflight (Kertsman et al., 2012). A decrease in myofascial tissue tone, spinal elongation, and the development of LBP does not necessarily correlate in time. Pain develops as a consequence of reduced muscle tone and an increase in spinal length. Thus, the sensation of pain requires a longer time to develop than physical changes. LBP observed in previous studies (Rukavishnikov et al., 2017; Tomilovskaya et al., 2020) has only peaked after 12–24 h h of unloading. This could suggest that time spent in the bath was insufficient for triggering sufficient physical changes to provoke more intense pain in the back. Nevertheless, DI appears to mimic the level, type, and distribution of LBP experienced in space to some extent.

### 4.4 Correlations

Spinal length increases correlated with middle thoracic (T9) myofascial tone reduction, but with no other m. *erector spinae* biomechanical and viscoelastic changes. However, the reduction in viscoelastic effects at 6 h may have contributed to the failure to observe other significant correlations. It should also be noted that such trends are at odds with the increase in LBP severity seen at 6 h compared to 1 h. However, the relationship with the evolution of stature is unknown.

Interestingly, a positive correlation was observed between changes in spinal length and LBP. The relationship between LBP and spinal lengthening reported by Hutchinson et al. (1995) was taken to suggest that stretching of the spinal and paraspinal muscles may lead to the development of LBP sensation in simulated and actual microgravity. However, no significant correlation between changes in creep and spinal elongation or reported pain was observed.

Muscle atrophy is a common sequelae of microgravity (LeBlanc et al., 1995; Sayson & Hargens, 2008; Sayson et al., 2013). Previous research has observed atrophy of the lumbar paraspinal muscles (m. *multifidus*, m. *erector spinae*, m. *quadratus lumborum*) in astronauts (LeBlanc et al., 1995; Chang et al., 2016). The spinal postural muscle atrophy likely becomes a primary musculoskeletal response to unloading due to a predominance of gravity-sensitive Type I muscle fibres in the deep and superficial m. *erector spinae* and m. *multifidus* (MacDonald et al., 2006). However, 6 h in DI was not sufficient to induce trunk muscle atrophy. Thus, further longer-term investigations of m. *erector spinae* (and other muscle) biomechanical and viscoelastic properties and their relationship to LBP development and vertebral column functionality are warranted.

## 5 Conclusion

This study demonstrates that DI-induced bilateral m. *erector spinae* tone, creep, and stiffness changes persist beyond 2 h—albeit tending to attenuate. Thus, whilst spinal elongation and LBP were induced, its relationship with the trunk myofascial tissue changes is complex and its role in LBP pathogenesis observed in real and simulated microgravity is yet to be determined. Further study is warranted with longer DI duration and additional assessment of IVD geometry and vertebral column stability. Correct quantitative assessment of trunk myofascial tissue changes will likely enhance the determination of astronauts’ predisposition to back pain or strong muscle atony in prolonged space flight. Experiments performed with DI may benefit the design and validation of specific countermeasures against the deterioration of back muscle biomechanical and viscoelastic properties induced by microgravity and hypokinesia.

## Data Availability

The original contributions presented in the study are included in the article/Supplementary Material, further inquiries can be directed to the corresponding author.
