# Supplementary material for: Dry immersion induced acute low back pain and its relationship with trunk myofascial viscoelastic changes
Source: Front Physiol. 2022 Oct 13;13:1039924. doi: 10.3389/fphys.2022.1039924 (PMC9606241; doi:10.3389/fphys.2022.1039924)
Supplement: Supplementary file 1 [file Table1.DOCX]

Supplementary Material

| Subject Number | Date | Age | Height (cm) | | | | Weight (kg) | | | |
| --- | --- | --- | --- | --- | --- | --- | --- | --- | --- | --- |
|  |  |  | Before DI | | After DI | | Before DI | | After DI | |
|  |  |  |  | |  | |  | |  | |
| **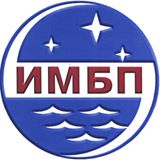** |  | | | | | | | | | |
|  | **Numeric Rating Scale (0-10)** | | **Left side of the back** | | | | **Right side of the back** | | | |
|  |  |  | Lumbar | Thoracic | | Cervical | Lumbar | Thoracic | | Cervical |
| **2h before DI** |  | |  |  | |  |  |  | |  |
| **1h DI** |  | |  |  | |  |  |  | |  |
| **6h DI** |  | |  |  | |  |  |  | |  |
| **30m after DI** |  | |  |  | |  |  |  | |  |
| **Notes** |  | | | | | | | | | |
|  |  | | | | | | | | | |
|  |  | | | | | | | | | |
|  |  | | | | | | | | | |

**Supplementary Figure 1.** Dry Immersion Data Collection Form


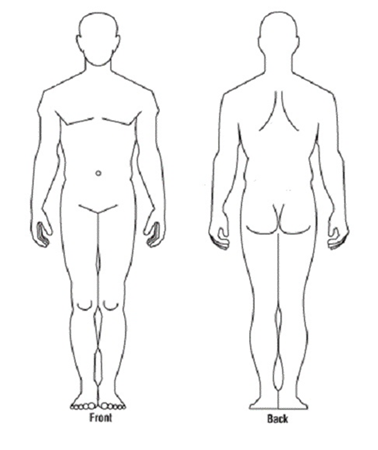


**Supplementary Figure 2.** Body Pain Map


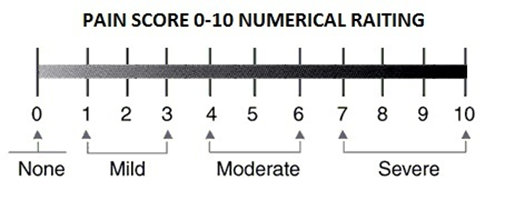


**Supplementary Figure 3.** Pain Numeric Rating Scale (Hawker et al., 2011).
